# Supplementary material for: Structural mechanisms of PIP2 activation and SEA0400 inhibition in human cardiac sodium-calcium exchanger NCX1
Source: bioRxiv. 2025 Mar 13:2024.12.05.627058. Originally published 2024 Dec 6. Preprint. [Version 2] doi: 10.1101/2024.12.05.627058 (PMC11643123; doi:10.1101/2024.12.05.627058)
Supplement: 1 [file NIHPP2024.12.05.627058V2-supplement-1.pdf]

bioRxiv preprint doi: https://doi.org/10.1101/2024.12.05.627058; this version posted March 13, 2025. The copyright holder for this preprint (which was not certified by peer review) is the author/funder, who has granted bioRxiv a license to display the preprint in perpetuity. It is made available under aCC-BY 4.0 International license.

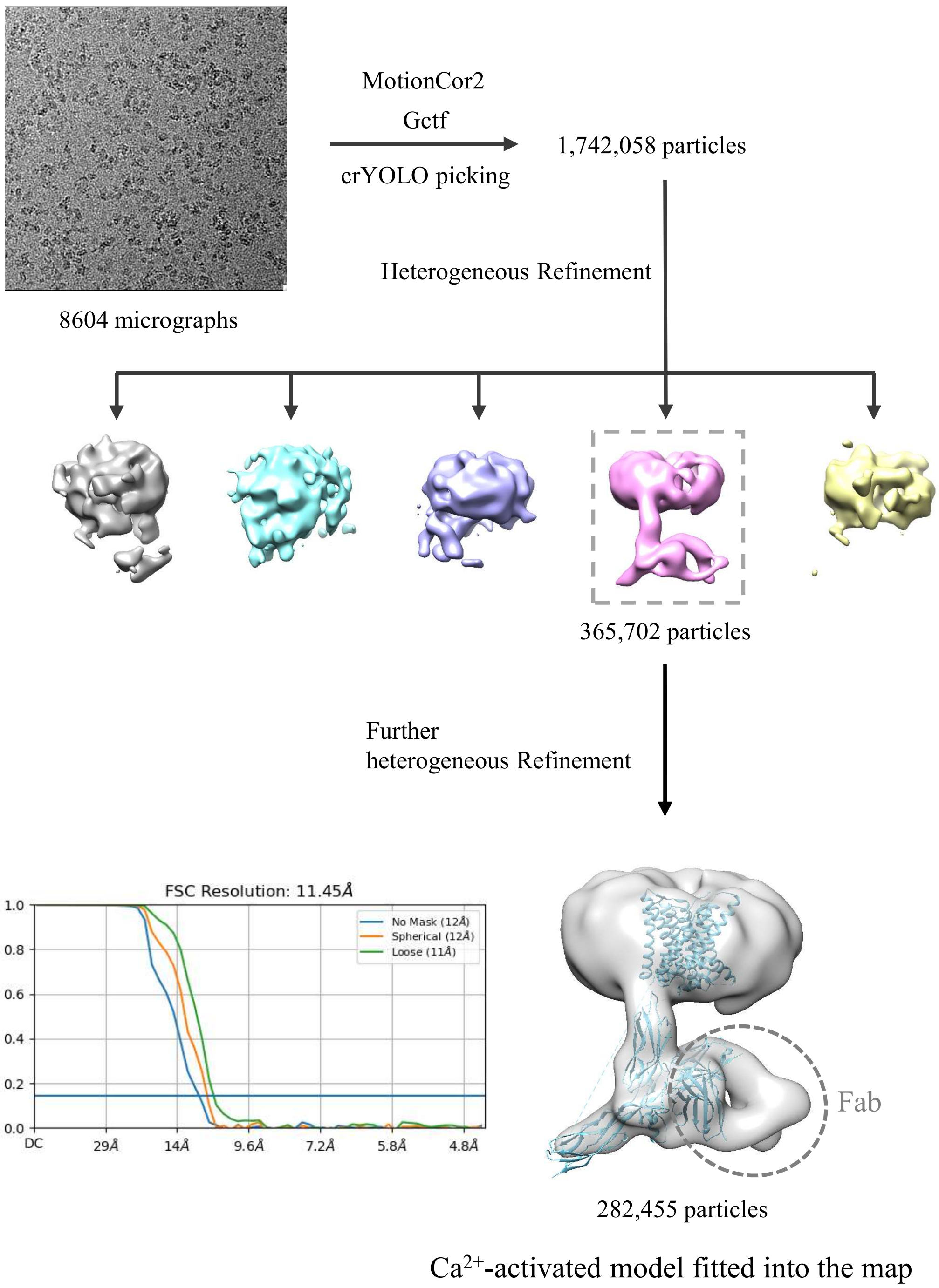

Fig. S1. Cryo-EM data processing of HsNCX1 in the presence of long-chain porcine brain PIP_2. The structural model of Ca^{2+}-activated HsNCX1 from a previous study (PDB 8SGT) was directly fitted into the low-resolution EM-map (~11.5 Å). The Fab fragment from a monoclonal antibody against NCX1 was used as a fiducial marker to facilitate the single-particle alignment.

bioRxiv preprint doi: https://doi.org/10.1101/2024.12.05.627058; this version posted March 13, 2025. The copyright holder for this preprint (which was not certified by peer review) is the author/funder, who has granted bioRxiv a license to display the preprint in perpetuity. It is made available under aCC-BY 4.0 International license.

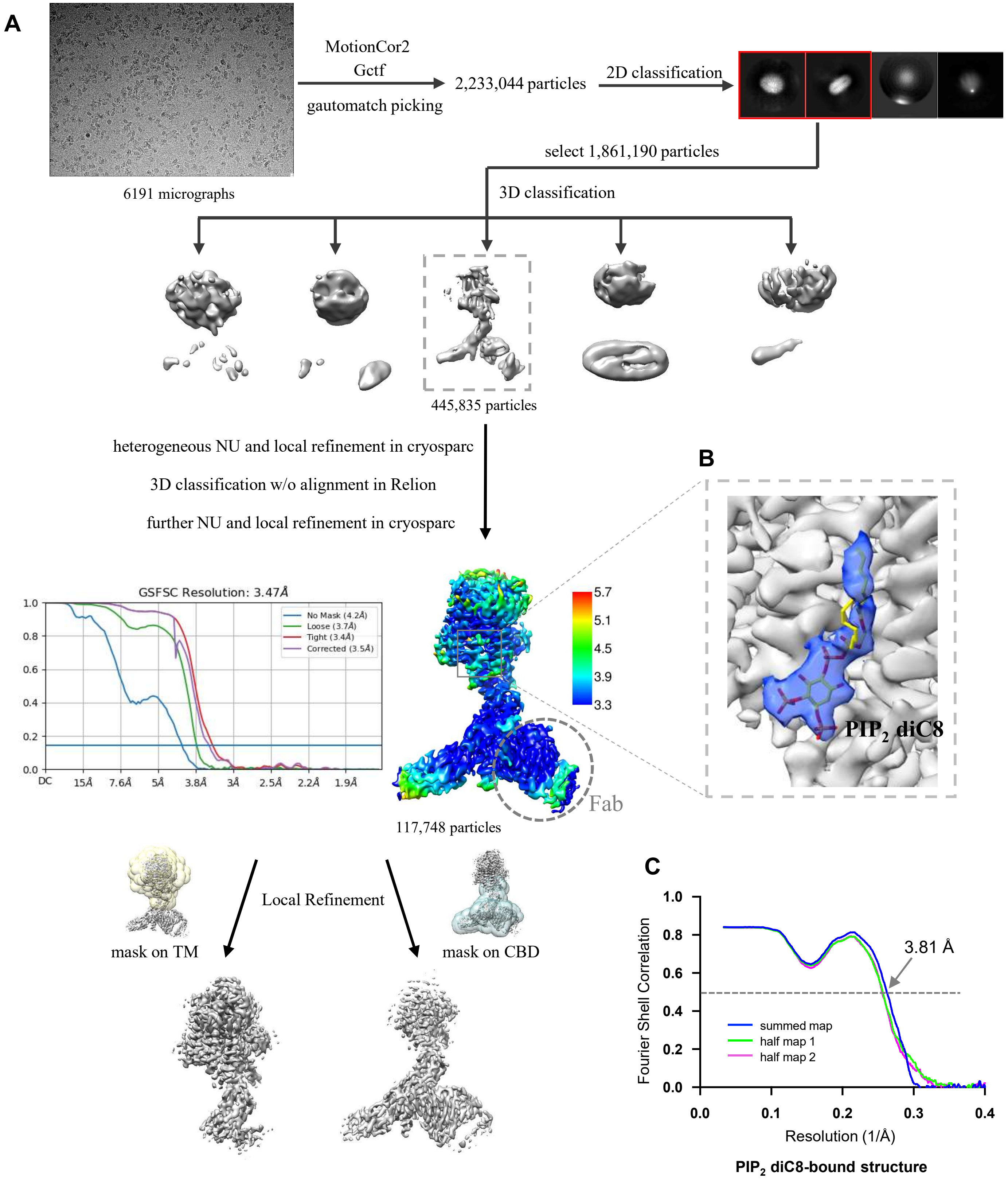

Fig. S2. Structure determination of HsNCX1-PIP$_2$ diC8 complex.
(A) Cryo-EM data processing of HsNCX1 in complex with short-chain PIP$_2$ diC8. The Fab fragment from a monoclonal antibody against NCX1 was used as a fiducial marker to facilitate the single-particle alignment.
(B) Zoomed-in view of density map of the bound PIP$_2$ diC8 contoured at the threshold level of 0.35 using ChimeraX.
(C) The Fourier shell correlation (FSC) curves for cross-validation between the maps and the models.

bioRxiv preprint doi: https://doi.org/10.1101/2024.12.05.627058; this version posted March 13, 2025. The copyright holder for this preprint (which was not certified by peer review) is the author/funder, who has granted bioRxiv a license to display the preprint in perpetuity. It is made available under aCC-BY 4.0 International license.

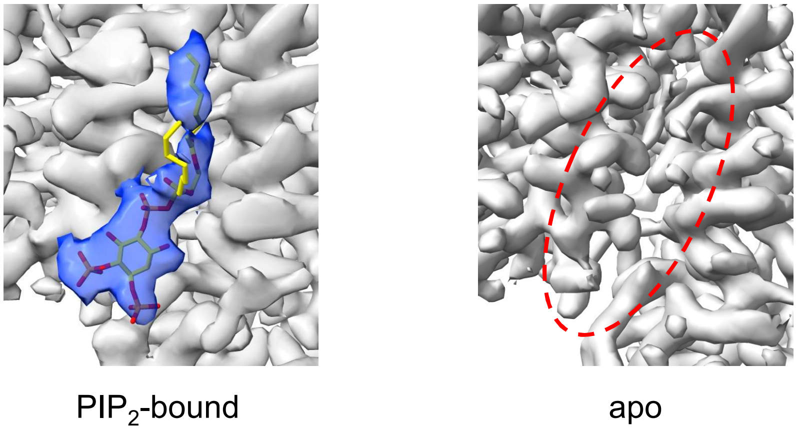

Fig. S3. Side-by-side comparison of the densities at the PIP_2-binding site between the PIP_2-bound structure (EMD-60921) and the apo structure (EMD-40457). The local-refined maps focused on the TM domain are used in the comparison. The density map of the bound PIP_2 is contoured at a threshold level of 0.35 using ChimeraX.

bioRxiv preprint doi: https://doi.org/10.1101/2024.12.05.627058; this version posted March 13, 2025. The copyright holder for this preprint (which was not certified by peer review) is the author/funder, who has granted bioRxiv a license to display the preprint in perpetuity. It is made available under aCC-BY 4.0 International license.

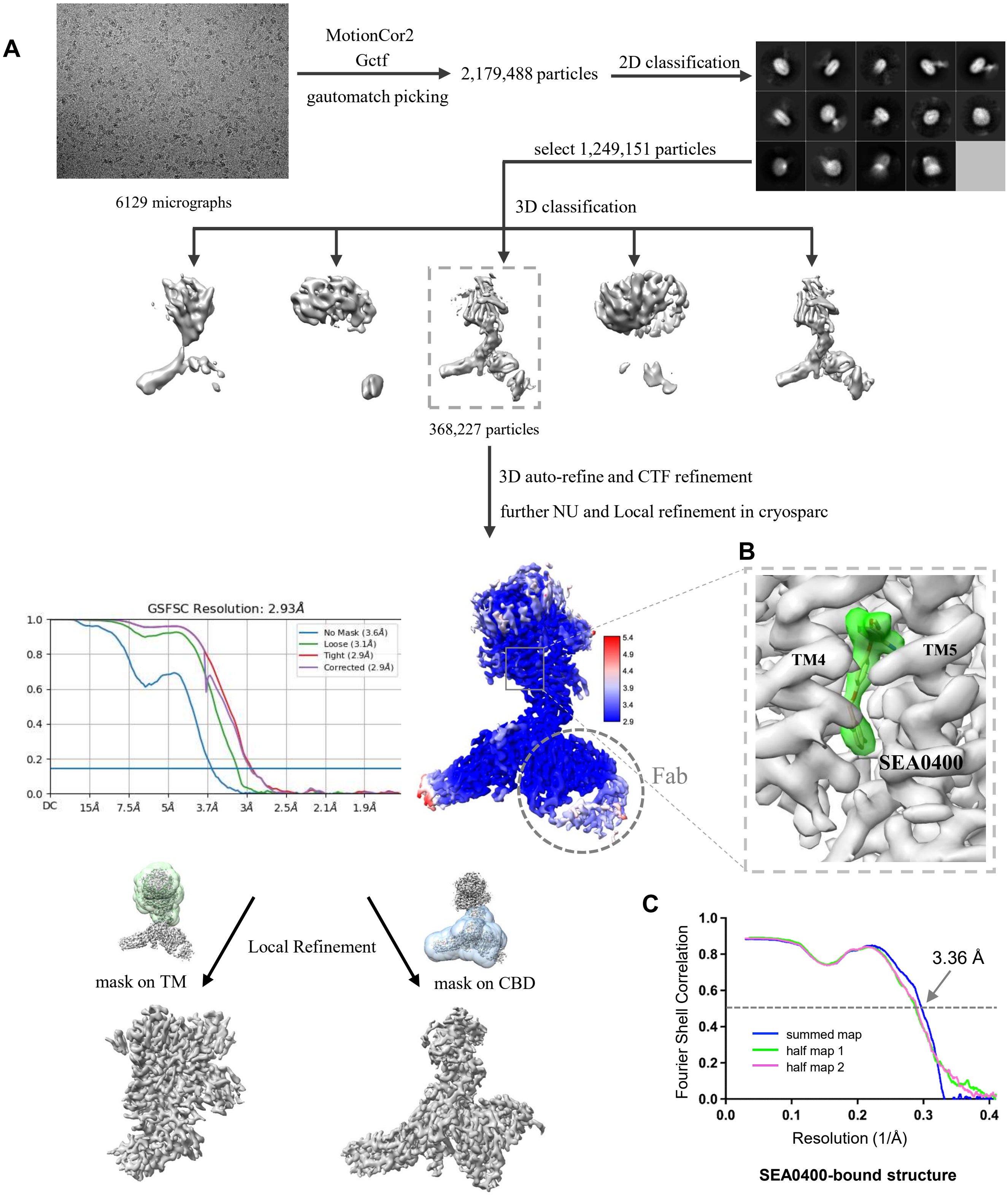

Fig. S4. Structure determination of HsNCX1-SEA0400 complex.
(A) Cryo-EM data processing scheme of HsNCX1 in complex with SEA0400 inhibitor. The Fab fragment from a monoclonal antibody against NCX1 was used as a fiducial marker to facilitate the single-particle alignment.
(B) Zoomed-in view of density map of the bound SEA0400 inhibitor contoured at the threshold level of 0.52 using ChimeraX.
(C) The Fourier shell correlation (FSC) curves for cross-validation between the maps and the models.

bioRxiv preprint doi: https://doi.org/10.1101/2024.12.05.627058; this version posted March 13, 2025. The copyright holder for this preprint (which was not certified by peer review) is the author/funder, who has granted bioRxiv a license to display the preprint in perpetuity. It is made available under aCC-BY 4.0 International license.

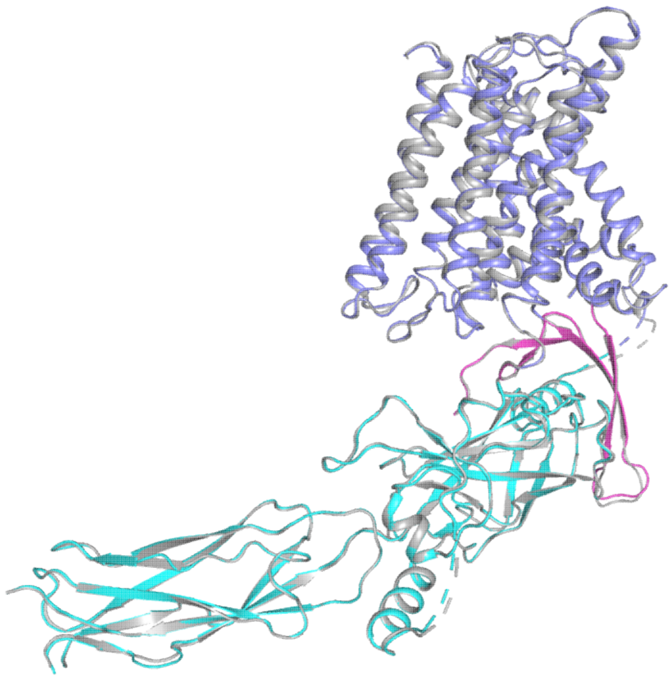

Fig. S5. Structural comparison between the apo (grey) and SEA0400-bound (color) HsNCX1.

bioRxiv preprint doi: https://doi.org/10.1101/2024.12.05.627058; this version posted March 13, 2025. The copyright holder for this preprint (which was not certified by peer review) is the author/funder, who has granted bioRxiv a license to display the preprint in perpetuity. It is made available under aCC-BY 4.0 International license.

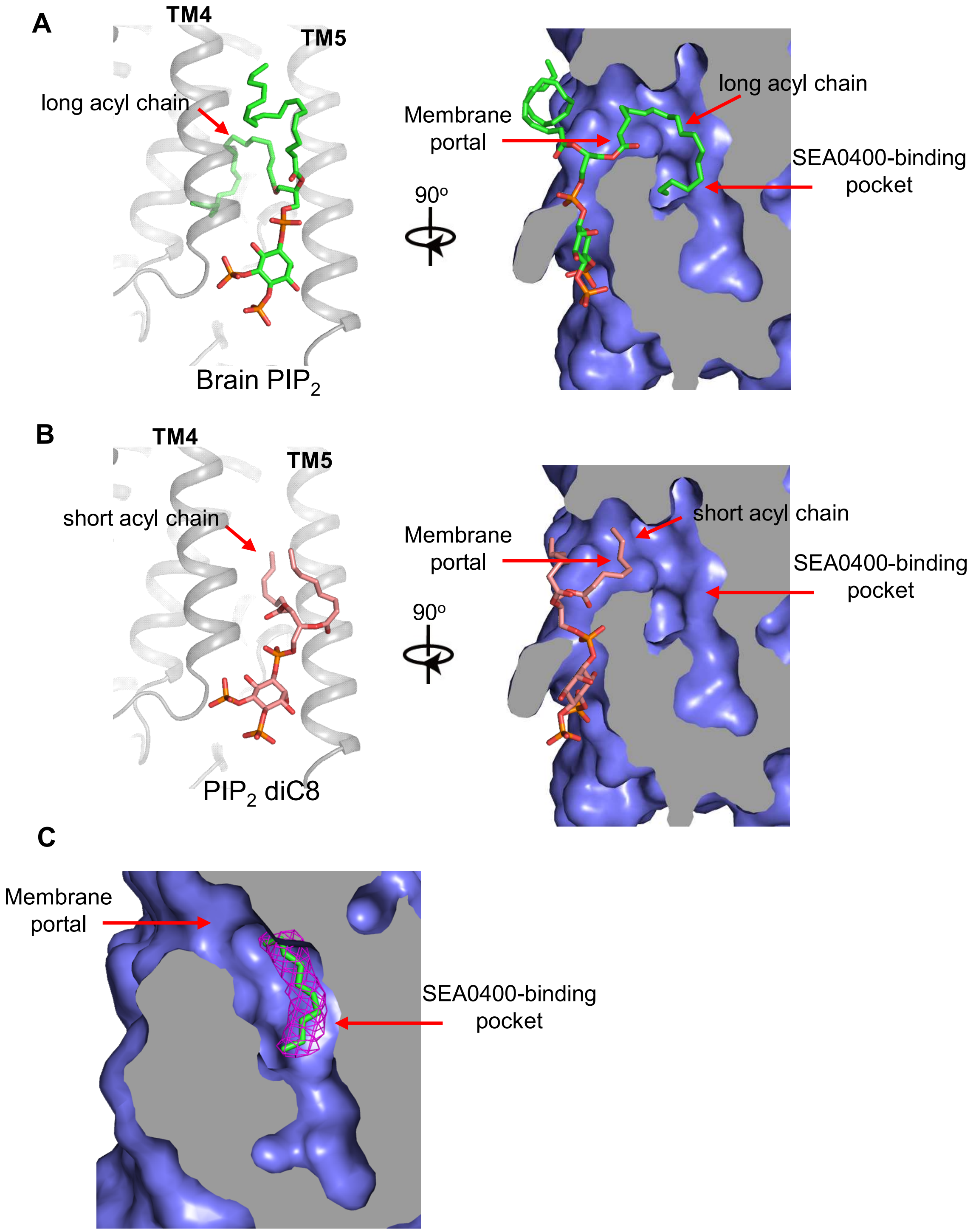

Fig. S6. Proposed structural basis underlying the different binding affinity between long and short-chain PIP_2.
(A) A docking model for native brain PIP_2 binding in NCX1 showing the insertion of its long acyl chain into the SEA0400-binding pocket.
(B) A docking model for short-chain PIP_2 diC8 binding in NCX1 showing that the SEA0400-binding pocket is not accessible to shorter acyl chain.
(C) The density (red mesh, contoured at 6σ) of an acyl chain likely from a native lipid is observed in the SEA0400-binding pocket of the apo NCX1 structure (EMD-40457, local-refined map at the TM domain).

bioRxiv preprint doi: https://doi.org/10.1101/2024.12.05.627058; this version posted March 13, 2025. The copyright holder for this preprint (which was not certified by peer review) is the author/funder, who has granted bioRxiv a license to display the preprint in perpetuity. It is made available under aCC-BY 4.0 International license.

Table S1. Cryo-EM data collection and model statistics.

**Sample preparation conditions**	25 mM Hepes pH 7.4,<br>200 mM NaCl<br>0.9 mM SEA0400	25 mM Hepes pH 7.4,<br>200 mM NaCl,<br>0.47 mM PI(4,5)P_2 diC8
	**SEA0400-bound state**<br>(EMD-40456,<br>PDB 8SGI)	**PI(4,5)P_2 diC8-bound state**<br>(EMD-60921,<br>PDB 9IV8)
**Data collection and processing**		
Magnification	105k	105k
Voltage (kV)	300	300
Electron exposure (e^-/Å^2)	60	60
Defocus range (μm)	-0.9 - -2.2	-0.9 - -2.2
Pixel size (Å)	0.83	0.84
Symmetry imposed	C1	C1
Initial particle images (no.)	1,249,151	2,233,044
Final particle images (no.)	368,227	117,748
Map resolution (Å)<br>FSC threshold	2.93<br>0.143	3.47<br>0.143
**Refinement**		
Initial model used<br>(PDB code)	8SGJ	8SGJ
Model resolution (Å)<br>FSC threshold	3.36<br>0.5	3.81<br>0.5
Model composition<br>Non-hydrogen atoms<br>Protein residues<br>Ligands	 7667<br>982<br>3: Na<br>6: Ca<br>1: H_2O<br>1: SEA0400	 5947<br>750<br>5: Ca<br>1: PI(4,5)P_2 diC8
B factors (Å^2)<br>Protein<br>Ligands	 66.21<br>58.55	 50.47<br>106.20
R.m.s. deviations<br>Bond lengths (Å)<br>Bond angles (°)	 0.005<br>0.696	 0.004<br>0.679
Validation<br>MolProbity score<br>Clashscore<br>Poor rotamers (%)	 1.36<br>5.24<br>0	 1.28<br>5.22<br>0
Ramachandran plot<br>Favored (%)<br>Allowed (%)<br>Disallowed (%)	 97.62<br>2.38<br>0	 98.24<br>1.76<br>0
